# Impact of BMI on the outcome of metastatic breast cancer patients treated with everolimus: a retrospective exploratory analysis of the BALLET study

**DOI:** 10.18632/oncotarget.27612

**Published:** 2020-06-09

**Authors:** Silvia P. Corona, Fabiola Giudici, Guy Jerusalem, Eva Ciruelos, Carla Strina, Marianna Sirico, Ottavia Bernocchi, Manuela Milani, Martina Dester, Nicoletta Ziglioli, Giuseppina Barbieri, Valeria Cervoni, Filippo Montemurro, Daniele Generali

**Affiliations:** ^1^ Department of Medicine, Surgery and Health Sciences, University of Trieste, Cattinara Hospital, Trieste, Italy; ^2^ Unit of Biostatistics, Epidemiology and Public Health, Department of Cardiac, Thoracic, Vascular Sciences and Public Health, University of Padua, Padua, Italy; ^3^ CHU Sart Tilman Liège and Liège University, Liège, Belgium; ^4^ Hospital Universitario 12 de Octubre, Madrid, Spain; ^5^ Multidisciplinary Unit of Breast Pathology and Translational Research, Cremona Hospital, Cremona, Italy; ^6^ Università Cattolica del Sacro Cuore, Roma, Italy; ^7^ Multidisciplinary Outpatient Oncology Clinic, Candiolo Cancer Institute, FPO-IRCCS, Candiolo, Italy

**Keywords:** BMI, weight, everolimus, metastatic breast cancer, outcomes

## Abstract

Introduction: Reliable biomarkers of response to mTOR inhibition are yet to be identified. As mTOR is heavily implicated in cell-metabolism, we investigated the relation between BMI variation and outcomes in metastatic breast cancer (mBC) patients treated with everolimus.

Results: we found a linear correlation between everolimus exposure duration and BMI/weight decrease. Patients exhibiting >2 kg weight loss or >3% BMI decrease from baseline at the end of treatment (EOT) had a statistically significant improvement in PFS. Interestingly, a similar BMI/weight decrease within the first 8 weeks of therapy identified patients at higher risk of progression.

Patients and methods: we performed a retrospective analysis of patients enrolled in the BALLET trial who progressed during the study. Primary end-point was progression-free survival (PFS). Secondary end-point was the identification of other predictors of response.

Conclusion: A >3% weight loss at EOT is associated with better outcome in mBC patients treated with everolimus. On the contrary, a significant early weight loss represents a predictor of poor survival and could therefore be used as an early negative prognostic marker. As PI3K-inhibition also converges onto mTOR, these findings might extend to patients treated with selective PI3K inhibitors and warrant further investigation

## INTRODUCTION

Activation of phosphatidylinositol 3-kinase (PI3K)—Mammalian Target of Rapamycin (mTOR)—is a with resistance to endocrine therapies [1, 2] and targeting PI3K-mTOR reverses this resistance. The BOLERO-2 study showed the efficacy of the combination of mTOR-inhibitor everolimus plus exemestane in patients with ER-positive/HER2-negative advanced breast cancer (BC) resistant to non-steroidal aromatase inhibitors (NSAIs), with a significant improvement in progression-free survival (PFS) in comparison to exemestane monotherapy (7.8 months versus 3.2 months) [3]. These findings led to FDA approval of the dual-blockade for the treatment of advanced or metastatic hormone-receptor positive BC which progressed after NSAIs.

Unfortunately, reliable biomarkers of response to mTOR targeted therapy are yet to be identified.

There is growing evidence that the PI3K-mTOR axis affects cellular metabolism. Weight loss and other metabolic side-effects are commonly observed in patients receiving PI3K and mTOR inhibitors [4–13]. Their relatively high prevalence makes these drug-induced adverse events good candidates for the role of surrogate biomarkers of everolimus efficacy [14–16].

Furthermore, recent studies suggest that BMI has prognostic value in metastatic breast cancer [17–19].

The BALLET study is a phase IIIb, expanded access, multicentre trial evaluating the safety of everolimus plus exemestane in patients with hormone receptor-positive, advanced or metastatic BC who progressed on prior NSAIs [20].

Here, we present the results of a retrospective, exploratory analysis evaluating the impact of BMI and weight variation on the outcome of a subgroup of patients participating in the BALLET trial and whose disease progressed during the study.

## RESULTS

As we wanted to investigate the correlation between BMI variation during treatment and risk of progression, only six-hundred-eighty-seven patients who progressed during the trial were included in this analysis. 635 patients (92.43%) had at least a BMI screening/baseline measurement and a corresponding post-baseline or EOT measurement.

**Figure 1:**
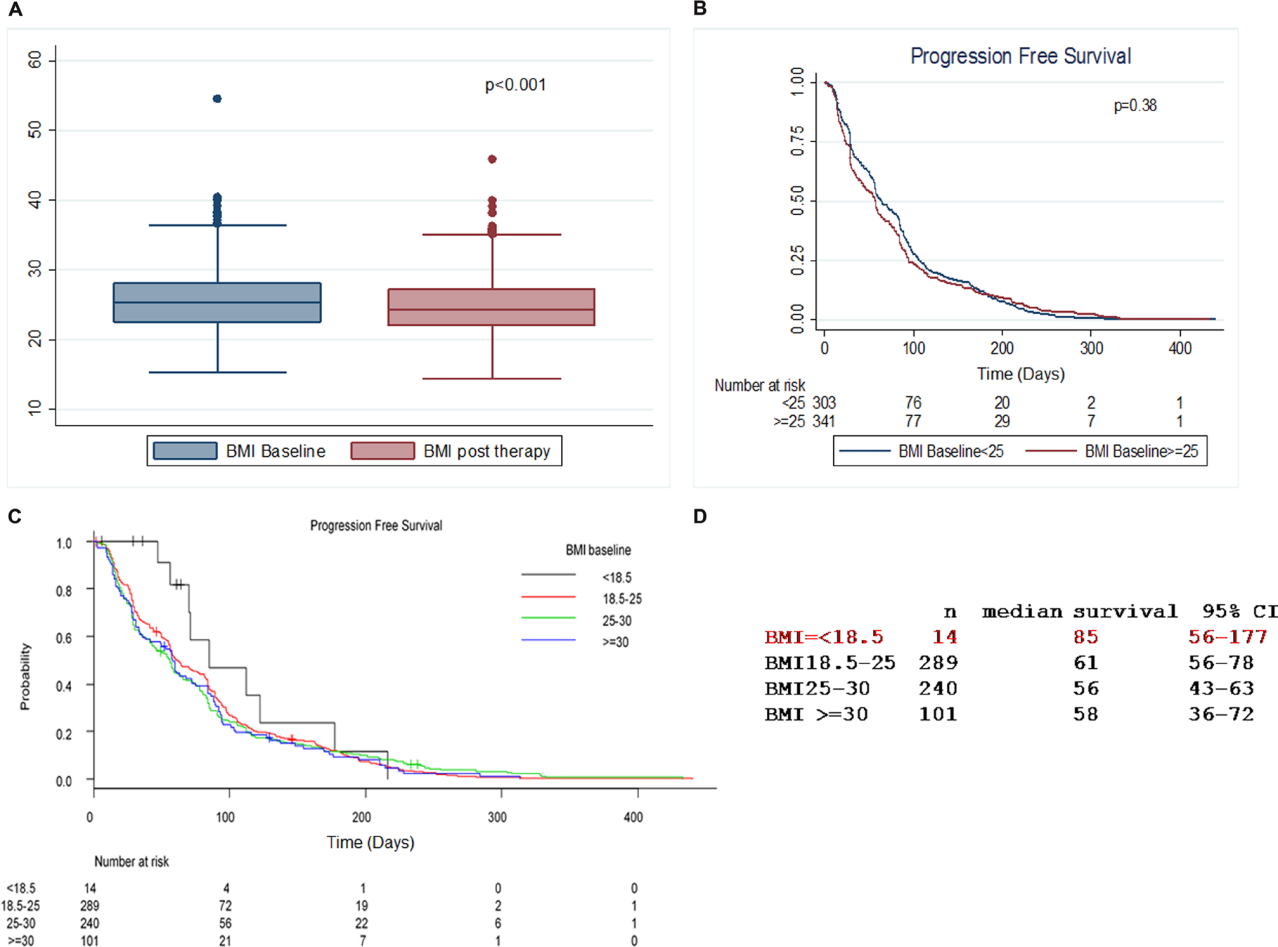
BMI changes during treatment with everolimus. (**A**) Statistically significant decrease in BMI at the end of therapy (EOT) in comparison to the baseline values. (**B**) Correlation between BMI values at baseline and progression-free survival (PFS). (**C**) Patients were stratified in 4 classes according to BMI values at baseline. Patients with BMI ≤ 18.5 Kg/m^2^ tend to show increased PFS (*p* = 0.45). Of note, only 14 patients have a BMI ≤ 18.5 Kg/m^2^ at baseline. (**D**) Median survival table in patients stratified in 4 classes according to BMI values at baseline.

We observed a statistically significant decrease in BMI at EOT in comparison to the baseline values (median BMI values, respectively, 24.29 versus 25.31, Figure 1A).

There was no correlation between BMI at baseline and PFS (*P* = 0.38, Figure 1). When we further stratified the patients by BMI categories, we observed an increased PFS in the group of women with lower BMI (Figure 1B). However, only 14 patients had a baseline BMI <18.5 kg/m^2^ and therefore this result should only be interpreted as a trend.

### Correlation between weight and exposure to everolimus

**Figure 2:**
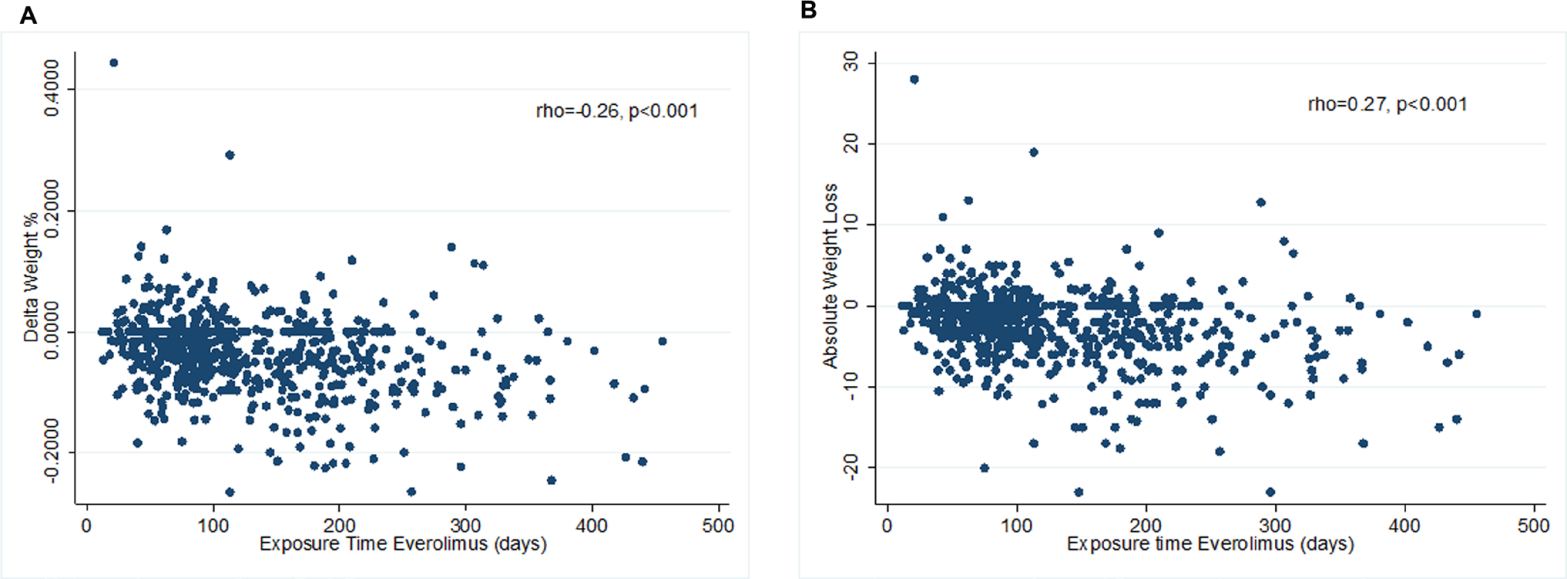
Correlation between duration of exposure to everolimus and weight change. (**A**) The weight change is expressed as percentage of weight lost from the initial weight. (**B**) The same correlation is observed when the weight loss is expressed as absolute weight in kilograms.

We found a linear correlation between everolimus exposure time and weight variation (Figure 2A and 2B). With the increase of the drug exposure time, we found a statistically significant increase in absolute weight loss in kg (rho = 0.27, *p* < 0.001) or percentage (rho = 0.26, *p* < 0.001).

After patient stratification according to the “Cancer-Associated Weight Loss” classification (see Materials and Methods), we found an association between everolimus exposure duration and weight loss severity (Supplementary Figure 1). The difference in exposure time according to the grade of weight loss was statistically significant (Kruskall Wallis test, *p* < 0.001). Median exposure time values increased proportionally with the increase of weight loss severity grade from 0 to 4.

### Correlation between BMI/weight changes and PFS

**Figure 3:**
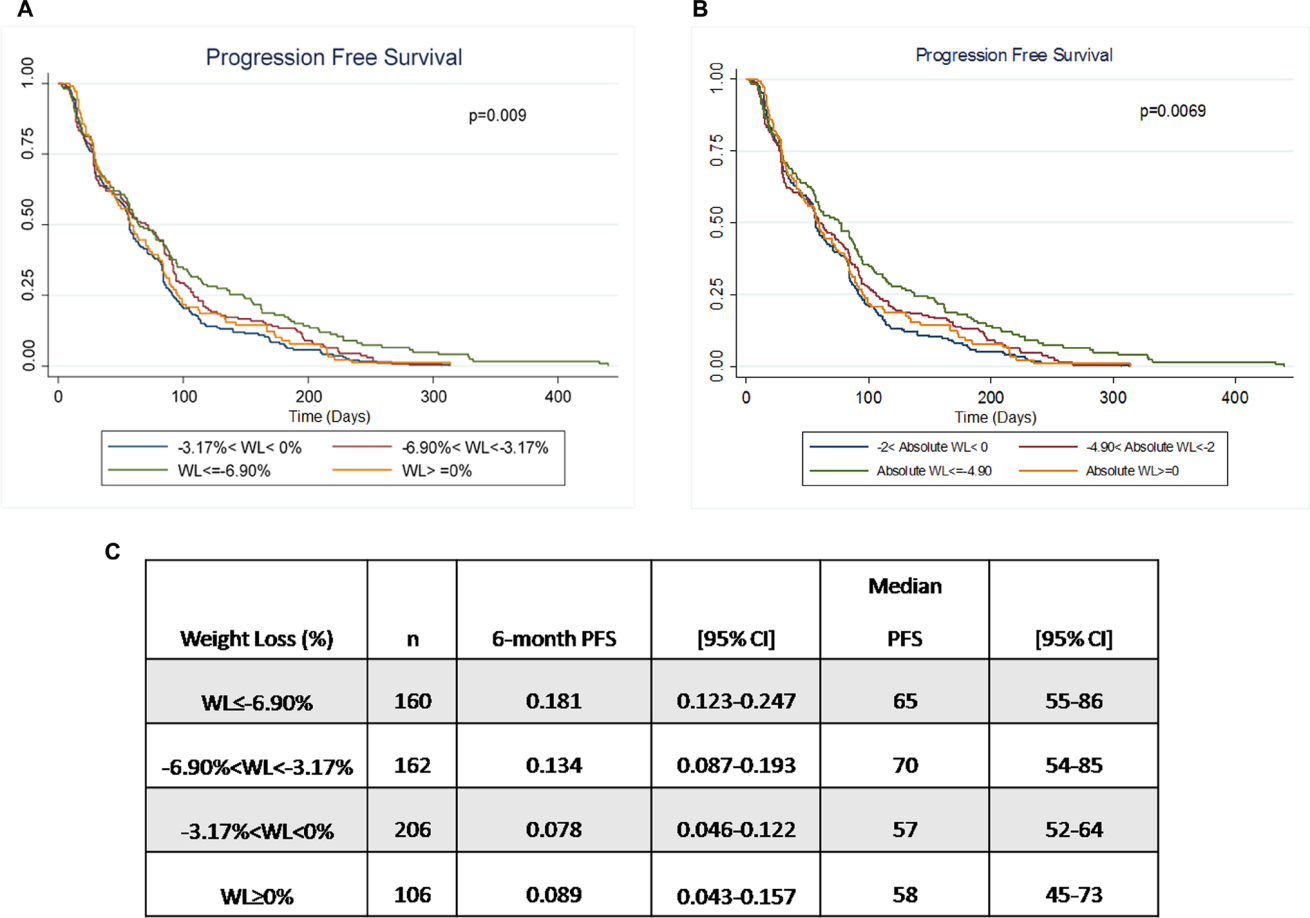
Correlation between weight change and PFS. (**A**) Patients were stratified according to weight loss expressed as percentage of initial weight loss during treatment. The two groups of patients who recorded the highest percentage of weight loss showed a better median PFS and the difference between groups was statistically significant (*p* < 0.009). (**B**) Patients were stratified according to the correspondent absolute weight loss expressed in kg. Patients with a weight loss of more than 4.90 kg show a statistically significant increase in median PFS (*p* < 0.0069). (**C**) Time to progression is significantly higher in patients with a percentage of weight loss > 6.90% (*p* = 0.00898).

We found a positive correlation between a weight loss >2 kg or 3.17% from baseline and the outcome, with a median PFS of around 70 (95%CI 55–86, *P* = 0.009) days versus 57 days on average for a weight loss of <2 kg or weight gain (Figure 3). PFS at 6 months was also statistically increased in the two groups recording the highest weight loss: 18.1% (95%CI 12.3–24.7) for the patients who lost more than 6.90% and 13.4% for the ones who lost between 3.17% and 6.90%, Figure 3C). In particular, after a post-hoc analysis, the two groups which showed the more significant difference in terms of PFS were <−6.90% versus −3.17% and 0% (see Supplementary Tables 1 and 2).

This tendency was confirmed after further stratification of patients according to the “Cancer-Associated Weight Loss” classification (Supplementary Figure 2). In particular, the Hazard Ratio shows inverse correlation with the weight loss grade: patients with grade 3 and 4 weight loss have a better prognosis (HR = 0.69) in comparison to inferior grades (Supplementary Figure 2).

### Correlation between BMI variation at 4/8 weeks and PFS

To investigate the potential predictive value of BMI decrease in this patient cohort, we analysed “early” weight variation, at 4 and 8 weeks of treatment. After exclusion of patients who progressed within 4 (190 patients) or 8 weeks (304 patients), the number of patients analysed was respectively 440 and 318.

**Figure 4:**
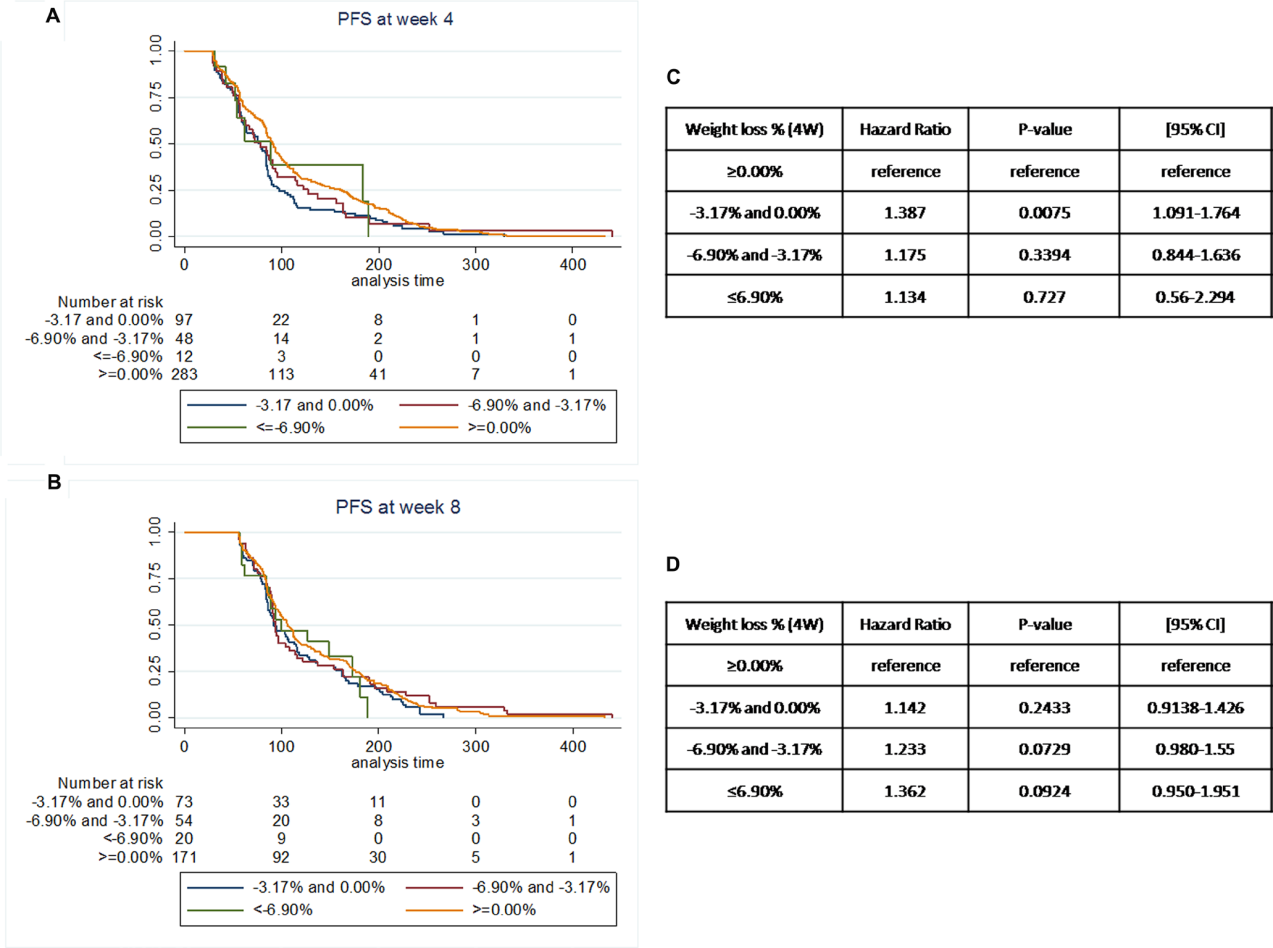
Landmark analysis of associations between progressive disease and weight loss percentage at landmark points (4 or 8 weeks of treatment). (**A**) Patients were stratified according to weight loss expressed as percentage of initial weight loss during treatment. All patients who recorded any degree of weight loss/BMI decrease after 4 weeks of treatment had worse prognosis in comparison to patients who gained weight. (**B**) A similar tendency was observed after 8 weeks of treatment, but in this case patients who recorded a weight loss/BMI decrease of more than 3.17% from the baseline value showed worse median PFS in comparison to patients who lost less or no weight. (**C**) PFS Hazard ratio (HR) according to the percentage of weight loss at 4 weeks. (**D**) PFS Hazard ratio (HR) according to the percentage of weight loss at 8 weeks.

Patients who recorded significant BMI variation at 4/8 weeks of treatment had worse prognosis, with this tendency being clearer at the 4-weeks time point (Figure 4). On the contrary, patients who gained weight showed a statistically significant increase in median PFS (*p* = 0.02, Log-Rank test). In particular, after a post-hoc analysis, the two groups which showed the more significant difference in terms of PFS were <−3.17% and 0% versus >0% (see Supplementary Tables 3 and 4).

**Figure 5:**
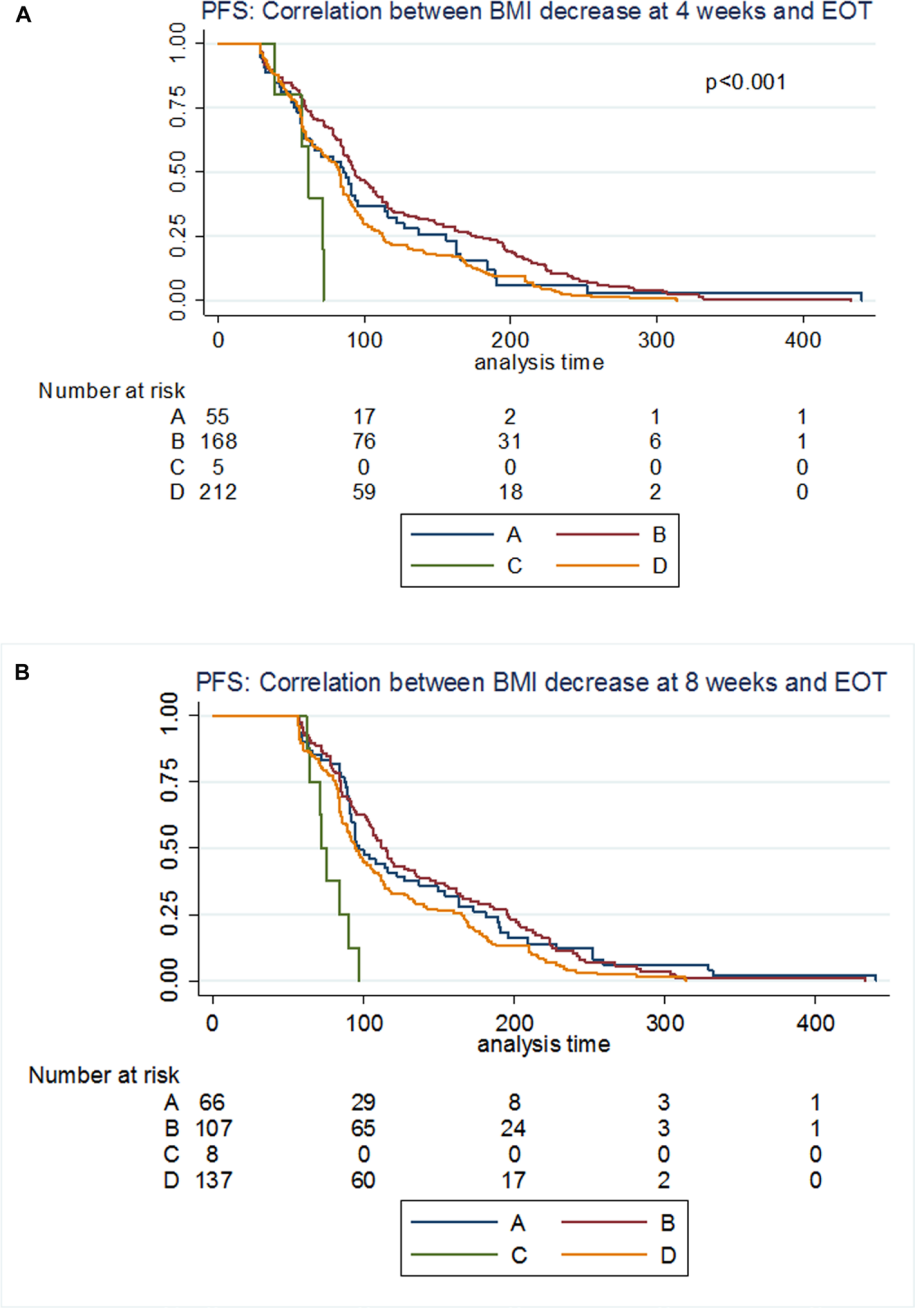
Correlation between weight/BMI decrease at 4/8/EOT and PFS. On the basis of the weight loss distribution at 4 or 8 weeks and at the end of treatment, we identified 4 categories of patients: A patients who lost more than 3.17% of their initial weight at 4 or 8 weeks as well as at the end of treatment; B patients who lost less than 3.17% of their initial weight at 4 or 8 weeks, but more than 3.17% by EOT; C. patients who lost more than 3.17% of their initial weight at 4 or 8 weeks, but less than 3.17% at EOT; D. patients who lost less than 3.17% of their initial weight at 4 or 8 weeks as well as by the EOT. We then correlated these 4 groups with the outcome expressed as PFS. (**A**) 4 weeks: EOT weight loss and PFS. (**B**) 8 weeks: EOT weight loss and PFS.

Furthermore, patients who recorded limited or no weight loss at 4 or 8 weeks, but lost >3.17% of their initial weight by EOT showed a statistically significant increase in both median- and 6-months PFS in comparison to all other 3 categories (median PFS 115 days, 95%CI 103–134, *P* < 0.001) (Figure 5). On the contrary, patients recording a significant weight loss at 4/8 weeks, but limited or no weight loss at EOT had the worst prognosis (median PFS 73.5 days, 95%CI 62-90).

## DISCUSSION

Weight loss is amongst the most commonly reported side effects of treatment with PI3K and mTOR inhibitors. 26.8% of patients randomized in the experimental arm of the SOLAR-1 study showed some degree of weight loss in comparison to only 2.1% of patients in the control group [4], with other studies reporting similar results [21, 22]. In our patient population, who progressed during the study, a much higher percentage of subjects treated with the drug experienced weight loss (76.85%), with 23.3% of our patients recording a loss of more than 6.90% of their baseline weight. This finding is in line with the results of another recent observational study investigating the predictive role of fasting glucose and BMI in breast cancer patients treated with the combination everolimus-exemestane [23].

Martin *et al*. developed a BMI-adjusted weight loss grading system for cancer patients, which also provides prognostic indications [24]. We used this classification as a tool to clearly identify significant differences in our population. However, while it helps defining the degrees of weight loss which have clinical implications for cancer patients, the classification does not differentiate according to cancer type and stage, nor does take into account other causes of weight loss beside cancer, such as co-morbidities and cancer-related treatments. Instead, it assumes that the cause is irrelevant as weight loss irremediably translates into metabolic dysregulation and worse prognosis, irrespective of the mechanisms behind it.

This assumption is simply not confirmed in our analysis, where everolimus-related weight loss seems to correlate with better prognosis and a PFS benefit.

Once the relationship between drug exposure time and weight was established, even in the presence of many confounding factors, the correlation between weight loss and progression free survival was explored. Women losing more than 3.17% of their initial BMI/weight during treatment with everolimus showed statistically significant improvement of 6 months-PFS in comparison to the others. Median PFS was also significantly higher in these patients compared to the ones who recorded limited or no weight loss (Figure 3).

While previous reports showed worse outcomes in patients with advanced cancer who developed significant weight loss [24, 25], we postulate that the weight loss observed in our patient population may be an on-target toxicity of everolimus and mTOR inhibitors, rather than expression of tumour-associated cachexia.

Of note, while cachexia and sarcopenia are extremely common in lung, gastrointestinal, prostate and head and neck cancers, the percentage of metastatic breast cancer patients who develop cancer-related wasting syndromes is reportedly small [26, 27]. However, diagnosis of cachexia cannot be made in the absence of muscle wasting [28, 29] and such occurrence is not normally investigated in the clinical setting. Instead, anthropometric measures are used as surrogates of muscle mass measures. Furthermore, a role for mTOR inhibitors in preventing and/or reversing tumour-associated cachexia through restoration of autophagy or reduction of IL-6 levels has been previously shown [30–32].

To investigate the predictive value of BMI decrease during everolimus treatment, we explored the relationship between early-stage weight loss and outcomes. Interestingly, patients who recorded a weight gain at 4 or 8 weeks, also showed an increased median PFS in comparison to all the others (Figure 4). This increase is more accentuated at 4 weeks, but still visible after 8 weeks of treatment. On the contrary, patients who recorded a significant weight loss in the first 4 weeks of treatment showed the worst prognosis. Notably, the number of patients who recorded a weight loss >6.90% from baseline was extremely low at both time points and therefore the results for this group of patients may not be as reliable. Nevertheless, these results were significantly strengthened by further analyses. We used the weight loss cut-off previously identified to stratify patients according to weight loss at 4 or 8 weeks and at EOT: patients who lost limited amount of weight (< 3.17%) or gained weightby 4 or 8 weeks, but also recorded a weight loss >3.17% by EOT, showed a statistically significant increase in median PFS in comparison to all other patients (Figure 5). Patients who recorded significant weight loss in the early stages and then limited weight loss or weight gain at EOT showed the worst prognosis.

Everolimus reaches steady state by 7 days [33] and noticeable changes in markers of activity of the drug are detected at least after 4 weeks of treatment [34]. Also, fast, unexplained weight loss is the hallmark of cancer cachexia, while weight variation by other causes is a metabolic multi-factorial response which requires time. Therefore, it is conceivable to think that any significant weight loss occurring between baseline and 4 weeks is synonymous of cancer-associated weight loss and not drug-induced, especially considering the patient population and progression risk. And in fact, in our analysis, the groups of patients who lost significant weight within 4 weeks of treatment (Figure 5A and 5C) in Figure 5) showed the worst prognosis in terms of median PFS (54 days A and 39 days C versus 78 days for group B, *P* = 0.00118).

Between 4 and 8 weeks of treatment, it becomes more difficult to distinguish between cancer related catabolism and drug effect, as the PFS curves and HRs tend to overlap (Figure 4B and 4D). This could be because a higher rate of patients may be experiencing wasting syndrome symptoms. In fact, 47% of the patients who recorded a weight loss of > 3.17% at EOT had already reported a quantitatively similar weight loss by week 8 (Supplementary Figure 3). These same patients do not do well if compared to other patients (median PFS of 115 days (B) versus 97 days (A), *P* = 0.0000906) (Figure 5B).

Significant decrease of BMI/weight in the early stages of everolimus treatment is associated with higher risk of progression and worse prognosis in our analysis, in accordance with previous reports [24, 35]. On the other hand, everolimus-associated weight loss recorded at EOT identifies a patient population gaining a clinical benefit from the mTOR inhibitor, which translates into a better outcome. As PI3K signalling converges onto mTOR, it is possible to hypothesize similar effects of PI3K selective inhibitors, such as Alpelisib.

Our study has some limitations: first of all, the retrospective nature of the data is prone to bias. Secondly, patients enrolled on the Ballet study were administered a combination of everolimus plus exemestane: metabolic effects of the aromatase inhibitor and pharmacological interaction cannot be excluded, as both drugs are metabolised in the liver. Also, the impact of tumour associated weight loss on this analysis cannot be accurately quantified despite all our effort, as it is difficult to distinguish amongst causes of weight loss. Furthermore, treatment interruptions length and adverse events seriousness varied across patients and could have affected the results. Finally, the impact of other potential confounding factors, such as other concurrent therapies or comorbidities cannot be excluded. Strengths of our analysis include the applied methodology and the use of stratification tools which account for both weight and BMI variations and are internationally recognised. Also, significant difference in survival outcomes is traditionally hard to demonstrate in heavily treated and advanced-stage patients.

Nevertheless, our study identified significant everolimus-associated weight loss as a positive prognostic factor which predicts PFS benefit in patients with advanced hormone-positive BC. On the other hand, early weight loss is associated with increased risk of disease progression and could be used as an early negative prognostic marker.

To our knowledge, our study is the first to report these findings. Our results underline the utility of BMI and weight information for cancer patients management. Stratification on the basis of these factors may help monitor treatment response in the clinical setting.

Prospective studies are needed to confirm and validate our results.

## MATERIALS AND METHODS

The BALLET study (EudraCT#2012-000073-23) recruited 2131 post-menopausal women with advanced or metastatic hormone receptor-positive breast cancer which recurred or progressed with non-steroidal aromatase inhibitors (NSAIs). The patients were enrolled irrespective of the number of prior lines of chemotherapy or other targeted treatments and NSAIs were not necessarily the last treatment these patients received.

Everolimus treatment was given in a 28-days cycle at a dose of 10 mg/day in combination with exemestane (25 mg/day). Treatment stopped in the case of disease progression, unacceptable toxicities, death, or local reimbursement of everolimus. Primary objective of the study was the assessment of the safety of the combination of everolimus plus exemestane. Secondary objectives included the evaluation of the grade 3 and 4 AEs severity.

As we were interested in the presence of a correlation between BMI variation and risk of disease progression, only patients who progressed during treatment were included in our analysis. EOT was always synonym of disease progression. 687 patients were evaluated. Weight measurements were recorded at baseline and in successive clinical assessments till the end or discontinuation of the study. The BMI was calculated as weight in kilograms divided by the square of height in meters (Kg/m^2^). A BMI between 18.5 and 24.9 was considered normal and a BMI ≥ 24.99 defined “overweight” [36]. As the height remains constant over time, we used weight or BMI interchangeably for our analyses.

We defined everolimus exposure time as the total number of days of administration, including restart after suspension. Weight variation was calculated by the formula ΔW= End of therapy (EOT) weight - BASELINE weight/BASELINE weight and expressed as percentage of weight loss.

In order to better classify weight loss severity in cancer patients, we used the classification of cancer-associated weight loss developed by Martin *et al*. [24]. Briefly, the grading system takes into account both weight loss percentage and BMI: the weight loss is expressed as function of the BMI measure at EOT and 5 degrees of increasing severity are identified as both percentages decrease (Table 1).

**Table 1:** The 5 × 5 matrix reported grading of weight loss (0–4) based on percentage weight loss and current body mass index in cancer patients (adapted from Martin *et a* *l*. 2015)

		**BMI (kg/m^2^)**				
		28 25 22 20				
**Weight Los s (%)**	2.5 6 11 15	0	0	1	1	3
		1	2	2	2	3
		2	3	3	3	4
		3	3	3	4	4
		3	4	4	4	4

Progression free survival (PFS), defined as the time between the start of everolimus and progression or death, was calculated in relation to BMI variation from baseline.

The study population was stratified in four subgroups according to the quartiles (25° percentile – median – 75° percentile) of absolute/percentage weight loss during treatment: difference baseline-EOT ≥0 kg or ≥0% (weight increase); difference baseline-EOT between 0 and 2 kg or between –3.17% and 0%; difference baseline-EOT between 2 and 4.90 kg or between –3.17% and –6.90%; difference baseline-EOT > 4.90 kg or ≤ −6.90%.

### Statistical analysis

Continuous variables were expressed as median and range (minimum-maximum), according to data distribution, after performing the Shapiro–Wilk test for normality. Categorical variables were expressed as absolute frequency and percentages and compared with Chi-Squared analysis. Baseline and EOT measurements of continuous variables were compared with Wilcoxon matched-pairs signed rank test. The relationship between everolimus exposure time and delta (Δ) weight loss was evaluated using the Spearman *rho*coefficient. Kruskall–Wallis test was applied to analyse everolimus exposure time stratified into 5 categories of weight loss severity according to Martin *et al*. [24]. Post-hoc tests, pair-wise comparisons using Mann–Whitney test, were conducted and corrected using the Holm method. PFS was estimated using the Kaplan–Meier approach and comparisons between survival distributions were performed with Log-Rank test. Since we were interested in the effect of weight/BMI loss on PFS and this is a time-dependent covariate, we used landmark analysis as the method of choice to avoid immortal time bias. Two separate analyses were performed at weeks 4 and 8, after beginning of therapy. Only patients who were alive and progression free at the time of the landmarks were included to avoid confounding factors. In order to evaluate the prognostic role of weight loss according to the “Classification of Cancer-Associated Weight Loss”, we used the univariate Cox regression model, with estimation of the Hazard Ratio (HR), after the proportional hazards assumption had been verified. All statistical analyses were performed using commercially available softwares (Stata/SE 14.1, Stata Corp LP, USA) and the R software version 3.5.0. All P values were calculated from 2-sided tests with 0.05 used as the significance level.

## SUPPLEMENTARY MATERIALS


