# Addendum: Impact of BMI on the outcome of metastatic breast cancer patients treated with everolimus: a retrospective exploratory analysis of the BALLET study

**DOI:** 10.18632/oncotarget.28317

**Published:** 2022-12-06

**Authors:** Silvia P. Corona, Fabiola Giudici, Guy Jerusalem, Eva Ciruelos, Carla Strina, Marianna Sirico, Ottavia Bernocchi, Manuela Milani, Martina Dester, Nicoletta Ziglioli, Giuseppina Barbieri, Valeria Cervoni, Filippo Montemurro, Daniele Generali

**Affiliations:** ^1^ Department of Medicine, Surgery and Health Sciences, University of Trieste, Cattinara Hospital, Trieste, Italy; ^2^ Unit of Biostatistics, Epidemiology and Public Health, Department of Cardiac, Thoracic, Vascular Sciences and Public Health, University of Padua, Padua, Italy; ^3^ CHU Sart Tilman Liège and Liège University, Liège, Belgium; ^4^ Hospital Universitario 12 de Octubre, Madrid, Spain; ^5^ Multidisciplinary Unit of Breast Pathology and Translational Research, Cremona Hospital, Cremona, Italy; ^6^ Università Cattolica del Sacro Cuore, Roma, Italy; ^7^ Multidisciplinary Outpatient Oncology Clinic, Candiolo Cancer Institute, FPO-IRCCS, Candiolo, Italy

**This article has an addendum:** Written informed consent was obtained from all patients. The study protocol was approved by an independent ethics committee or institutional review board at each site.

Original article: Oncotarget. 2020; 11:2172–2181. 2172-2181. https://doi.org/10.18632/oncotarget.27612

