# Supplementary material for: Impact of BMI on the outcome of metastatic breast cancer patients treated with everolimus: a retrospective exploratory analysis of the BALLET study
Source: Oncotarget. 2020 Jun 9;11(23):2172–81. doi: 10.18632/oncotarget.27612 (PMC7289535; doi:10.18632/oncotarget.27612)
Supplement: Supplementary file 1 [file oncotarget-11-2172-s001.pdf]

Impact of BMI on the outcome of metastatic breast cancer patients treated with everolimus: a retrospective exploratory analysis of the BALLET study

SUPPLEMENTARY MATERIALS

A

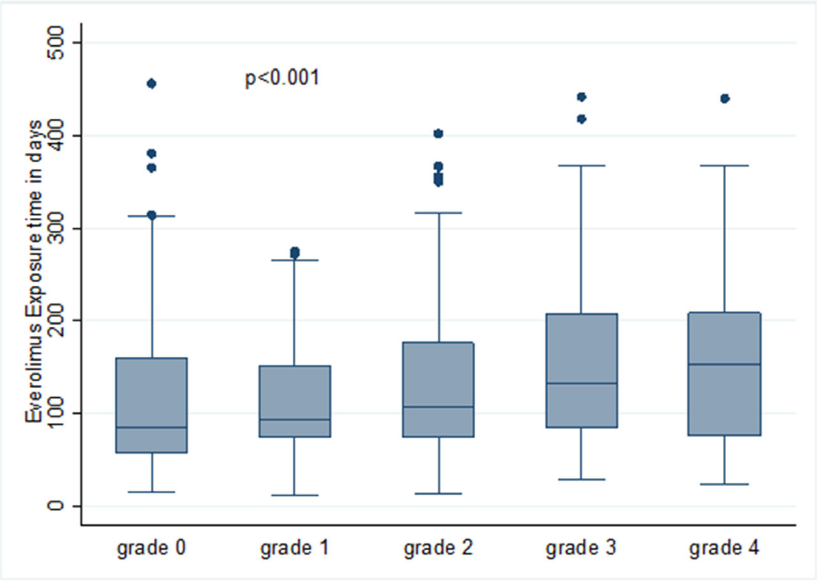

B

Grade	Median	min	max	N
grade 0	85	15	456	131
grade 1	93	12	275	156
grade 2	107.5	13	402	118
grade 3	132.5	28	442	140
grade 4	152.5	24	440	50

Supplementary Figure 1: Association between duration of exposure to everolimus and severity of weight loss according to the Classification of Cancer-Associated Weight Loss [24]. (**A**) Association between everolimus exposure time and grade of weight loss severity according to the "Classification of Cancer-Associated Weight Loss". (**B**) Median exposure time according to the grade of weight loss: the values increase proportionally with the increase of weight loss severity grade from 0 to 4.

A

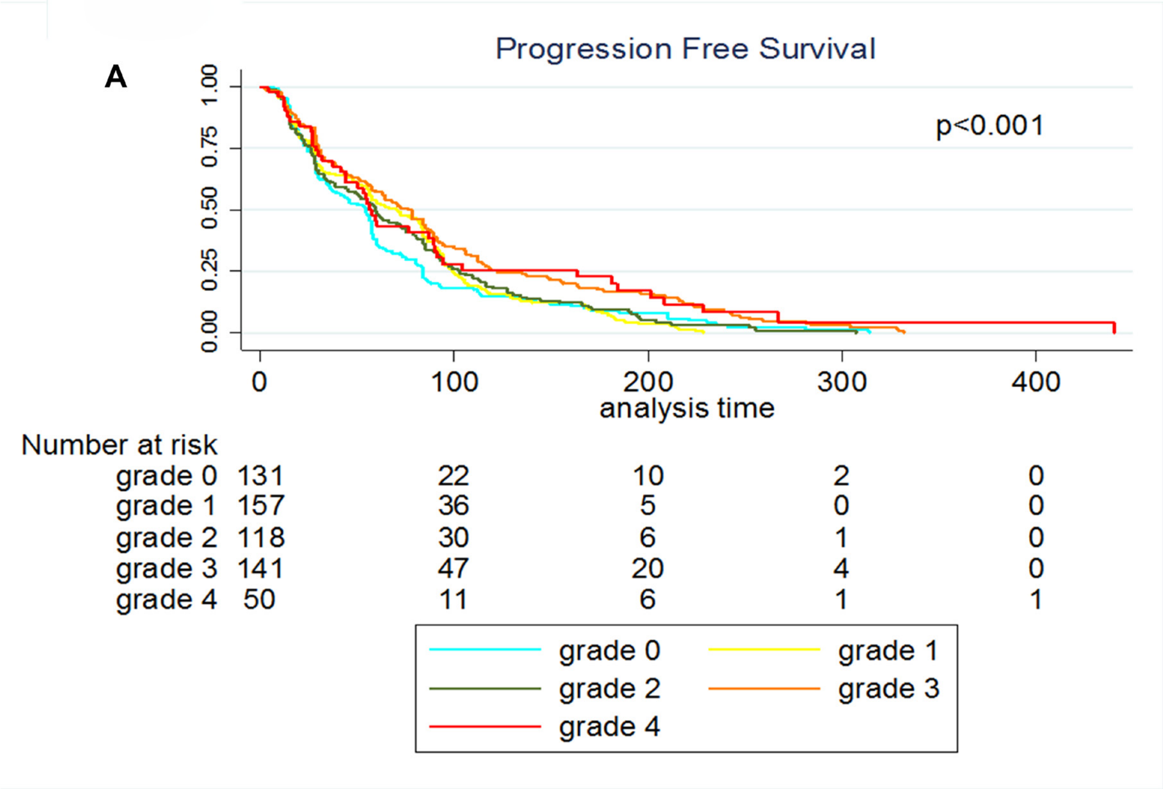

B

Weight loss Grade	Hazard Ratio	P-value	[95% CI]
Grade 0	reference	reference	reference
Grade 1	0.93	0.534	0.73-1.18
Grade 2	0.91	0.472	0.71-1.17
Grade 3	0.69	0.002	0.53-0.87
Grade 4	0.69	0.041	0.48-0.99

Supplementary Figure 2: Correlation between classes of weight loss according to the classification of cancer-associated weight loss and PFS. (**A**) Patients were stratified according to the BMI-adjusted weight loss percentage and the PFS measured. There is a statistically significant difference in PFS between grades of weight loss and weight loss grades 2 and 3 show the best outcome ($p <$ 0.001). (**B**) PFS Hazard ratio (HR) according to the grades of weight loss. Grade 3 and 4 show a HR = 0.69 versus 0.93 of grade 1 and 0.91 of grade 2. These differences are statistically significant.

A

Δ weight loss % at EOT	Δ weight loss % at 4 weeks<br><-3.17%	>-3.17%	Tot
<-3.17%	82 (26.89%)	223 (73.11%)	305 (51.09%)
≥ -3.17%	9 (3.08%)	283 (96.92%)	292 (48.91%)
Total	91 (15,24%)	506 (84.76%)	597 (100%)

B

Δ weight loss % at EOT	Δ weight loss % at 8 weeks<br><-3.17%	>-3.17%	Tot
<-3.17%	134 (46.85%)	152 (53.15%)	286 (54.166%)
≥ -3.17%	16 (6.61%)	226 (93.39%)	242 (45.833%)
Total	150 (28.41%)	378 (71.59%)	528 (100%)

Supplementary Figure 3: Distribution of weight loss/BMI decrease at 4 or 8 weeks of treatment according to the weight/BMI variation recorded at the end of treatment. (**A**) Considering the significance of a weight loss/BMI decrease of more than 3.17% in our outcome analysis, we used the same cut-off to analyse the distribution of weight/BMI variation in our study population after 4 weeks and at the end of treatment. Almost 27% of the patients who recorded a weight/BMI decrease higher than 3.17% at end of treatment had already a weight loss of more than 3.17% at the 4 weeks time point. (**B**) Distribution of weight/BMI variation after 8 weeks and at the end of treatment. Almost 47% of the patients who recorded a weight/BMI decrease higher than 3.17% at end of treatment had already a weight loss of more than 3.17% at the 8 weeks time point.

Supplementary Table 1: Multiple log-rank tests between pairs of weight loss (%) groups: adjusted *p*-value for multiple testing correction (Holm-Method)(see Figure 3A)

Weight loss groups		post-hoc *p*-value	*p* overall
<–6.90%	–6.90% and –3.17%	0.18	
**<–6.90%**	**–3.17% and 0%**	**0.001***	
<–6.90%	>0%	0.13	0.009
–6.90% and –3.17%	–3.17% and 0%	0.18	
–6.90% and –3.17%	>0%	0.51	
–3.17% and 0%	>0%	0.51	

Supplementary Table 2: Multiple log-rank tests between pairs of groups of absolute weight loss: adjusted *p*-value for multiple testing correction (Holm-Method) (see Figure 3B)

Absolute weight loss groups		post-hoc *p*-value	*p* overall
≤–4.90	–4.90 and 2	0.076	
**≤–4.90**	**–2 and 0**	**0.0069***	
≤–4.90	>0	0.076	0.00692
–4.90 and –2	–2 and 0	0.30	
–4.90 and –2	>0	0.688	
<2 and 0	>0	0.58	

Supplementary Table 3: Multiple log-rank tests between pairs of weight loss (%) groups: adjusted *p*-value for multiple testing correction (Holm-Method) (see Figure 4A)

Weight loss groups		post-hoc *p*-value	*p* overall log-rank test
<–6.90%	–6.90% and –3.17%	0.72	
<–6.90%	–3.17% and 0%	0.72	
<–6.90%	>0%	0.72	
–6.90% and –3.17%	–3.17% and 0%	0.72	
–6.90% and –3.17%	>0%	0.72	0.05
**–3.17% and 0%**	**>0%**	**0.03***	

Supplementary Table 4: Multiple log-rank tests between pairs of Weight loss (%) groups: adjusted *p*-value for multiple testing correction (Holm-Method) (see Figure 4B)

Weight loss groups		post-hoc *p*-value	*p* overall log-rank test
<–6.90%	–6.90% and –3.17%	0.90	
<–6.90%	–3.17% and 0%	0.90	
<–6.90%	>0%	0.90	
–6.90% and –3.17%	–3.17% and 0%	0.90	
–6.90% and –3.17%	>0%	0.90	0.53
–3.17% and 0%	>%	0.86	
